# *In Vivo* Molecular Imaging of the Efficacy of Aminopeptidase N (APN/CD13) Receptor Inhibitor Treatment on Experimental Tumors Using ^68^Ga-NODAGA-c(NGR) Peptide

**DOI:** 10.1155/2021/6642973

**Published:** 2021-03-10

**Authors:** Adrienn Kis, Noémi Dénes, Judit P. Szabó, Viktória Arató, Lívia Beke, Orsolya Matolay, Kata Nóra Enyedi, Gábor Méhes, Gábor Mező, Péter Bai, István Kertész, György Trencsényi

**Affiliations:** ^1^Division of Nuclear Medicine and Translational Imaging, Department of Medical Imaging, Faculty of Medicine, University of Debrecen, Nagyerdei St. 98, H-4032 Debrecen, Hungary; ^2^Doctoral School of Clinical Medicine, Faculty of Medicine, University of Debrecen, Nagyerdei St. 98, H-4032 Debrecen, Hungary; ^3^Gyula Petrányi Doctoral School of Allergy and Clinical Immunology, Faculty of Medicine, University of Debrecen, Nagyerdei St. 98, H-4032 Debrecen, Hungary; ^4^Department of Pathology, Faculty of Medicine, University of Debrecen, Nagyerdei St. 98, H-4032 Debrecen, Hungary; ^5^Eötvös Loránd University, Faculty of Science, Institute of Chemistry, Budapest, Hungary; ^6^MTA-ELTE, Research Group of Peptide Chemistry, Hungarian Academy of Sciences, Eötvös L. University, Budapest, Hungary; ^7^Department of Medical Chemistry, University of Debrecen, Nagyerdei St. 98, H-4032 Debrecen, Hungary; ^8^MTA-DE Lendület Laboratory of Cellular Metabolism, Debrecen, Hungary; ^9^Research Center for Molecular Medicine, University of Debrecen, Nagyerdei St. 98, H-4032 Debrecen, Hungary

## Abstract

**Introduction:**

The aminopeptidase N (APN/CD13) receptor plays an important role in the neoangiogenic process and metastatic tumor cell invasion. Clinical and preclinical studies reported that bestatin and actinonin are cytotoxic to APN/CD13-positive tumors and metastases due to their APN/CD13-specific inhibitor properties. Our previous studies have already shown that ^68^Ga-labeled NGR peptides bind specifically to APN/CD13 expressing tumor cells. The APN/CD13 specificity of ^68^Ga-NGR radiopharmaceuticals enables the following of the efficacy of antiangiogenic therapy with APN/CD13-specific inhibitors using positron emission tomography (PET). The aim of this *in vivo* study was to assess the antitumor effect of bestatin and actinonin treatment in subcutaneous transplanted HT1080 and B16-F10 tumor-bearing animal models using ^68^Ga-NODAGA-c(NGR).

**Materials and Methods:**

Three days after the inoculation of HT1080 and B16-F10 cells, mice were treated with intraperitoneal injection of bestatin (15 mg/kg) or actinonin (5 mg/kg) for 7 days. On the 5^th^ and 10^th^ day, *in vivo* PET scans and *ex vivo* biodistribution studies were performed 90 min after intravenous injection of 5.5 ± 0.2 MBq^68^Ga-NODAGA-c(NGR).

**Results:**

Control-untreated HT1080 and B16-F10 tumors were clearly visualized by the APN/CD13-specific ^68^Ga-NODAGA-c(NGR) radiopharmaceutical. The western blot analysis also confirmed the strong APN/CD13 positivity in the investigated tumors. We found significantly (*p* ≤ 0.05) lower radiopharmaceutical uptake after bestatin treatment and higher radiotracer accumulation in the actinonin-treated HT1080 tumors. In contrast, significantly lower (*p* ≤ 0.01) ^68^Ga-NODAGA-c(NGR) accumulation was observed in both bestatin- and actinonin-treated B16-F10 melanoma tumors compared to the untreated-control tumors. Bestatin inhibited tumor growth and ^68^Ga-NODAGA-c(NGR) uptake in both tumor models.

**Conclusion:**

The bestatin treatment is suitable for suppressing the neoangiogenic process and APN/CD13 expression of experimental HT1080 and B16-F10 tumors; furthermore, ^68^Ga-NODAGA-c(NGR) is an applicable radiotracer for the *in vivo* monitoring of the efficacy of the APN/CD13 inhibition-based anticancer therapies.

## 1. Introduction

Angiogenesis—the new blood vessel formation from a preexisting capillary system—plays an important role in different physiological processes such as wound healing [1] and the action of the female reproduction system [2], but it can emerge in malignant processes such as psoriasis [3, 4], rheumatoid arthritis [5], retinopathies [6], and cancers [7, 8]. During the angiogenic process, several receptors and molecules (e.g., VEGF, integrins, and APN/CD13) appear in the cell surface, which provide opportunities to detect and treat malignant tumors [9–12]. Among these angiogenic molecules, aminopeptidase N (APN/CD13) showed a strong correlation with tumor-associated neoangiogenesis [13–15]. APN/CD13 is a 160 kDa weighted and glycosilated, zinc-dependent transmembrane ectopeptidase. It has three main functions: enzyme, receptor, and signaling molecule [16]. As an enzyme, it plays an important role in peptide cleavage, such as angiotensins, kinins, enkephalins, cytokines, and chemokines. Furthermore, APN/CD13 participates in extracellular matrix protein degradation, which facilitates tumor cell invasion and migration. As a receptor, APN/CD13 is involved in endocytosis during viral infection; moreover, as a signaling molecule, it attends in adhesion, phagocytosis, and angiogenic processes [16]. APN/CD13 is physiologically expressed in the epithelial cells of the liver, intestine, and kidney and in the synaptic membranes and pericytes of the central nervous system [17]. Several studies reported that APN/CD13 is overexpressed in the endothelial cells of tumor vasculature and in several solid tumors, such as melanoma [18, 19], prostate carcinoma [20], lung cancer [21], pancreas adenocarcinoma [22], ovarian cancer [23], breast cancer [13], colon cancer [24], thyroid cancer [25], and fibrosarcomas [26]. Due to its elevated expression, APN/CD13 was reviewed as an important clinical marker in several inflammatory diseases and malignant cancers [22, 27, 28], and it has been considered as a suitable target for anticancer and anti-inflammatory therapy [29–32]. In antiangiogenesis therapy, the most frequently administered natural variants of APN/CD13 inhibitors are actinonin, amastatin, bestatin, phebestin, probestin, and curcumin, most of which are originated from bacteria or plants [30]. Bestatin is a well-known and potent APN/CD13 inhibitor which has already been investigated by several authors in *in vitro* and *in vivo* studies. Bestatin, due to its competitive, reversible protease inhibitor properties, has an antiangiogenic effect through the inhibition of APN/CD13's activity in numerous tumors (e.g., murine colon adenocarcinoma and myeloid leukemia [33], human promyelocytic leukemia [34], human choriocarcinoma [35], murine melanoma [36, 37], murine lung carcinoma [37], human prostate cancer [38], and human fibrosarcoma [39]). Moreover, in the field of radiotherapy, bestatin can enhance the radiosensitivity of human cervical cancer [40] and human renal cell carcinoma [41]. Actinonin—as a peptide deformylase inhibitor—is also an effective APN/CD13 inhibitor which has antiproliferative impact on human promyelocytic leukemia, human myeloid leukemia, and signalized antitumor effect on murine leukemia [42, 43].

Based on the information mentioned above, *in vivo* positron emission tomography (PET) can be a useful methodology to monitor the antiangiogenic therapeutic effect of actinonin and bestatin using APN/CD13-specific NGR-based radiopharmaceuticals. Our previous studies have already demonstrated that the ^68^Ga-labeled NGR peptide sequence (cKNGRE-NH_2_) specifically binds to APN/CD13 of experimental tumors [44, 45]. The aim of this present study was to investigate *in vivo* the effect of bestatin and actinonin treatment on APN/CD13 expression of HT1080 and B16-F10 tumors using APN/CD13-specific ^68^Ga-NODAGA-c(NGR) radiopharmaceutical and PET imaging.

## 2. Materials and Methods

### 2.1. Chemicals

All reagents were obtained from commercial suppliers and used without further purification. Cell culture media (Gibco™), FBS (Gibco™), antimycotic-antibiotic solution (Gibco™), MEM vitamin solution (Gibco™), and MEM nonessential amino acid solution (Gibco™) were obtained from Thermo Fisher Scientific Inc., USA. Actinonin and bestatin (Cayman Chemical Company) were purchased from VWR Ltd., Hungary. The NODAGA-NHS ester was purchased from CheMatech (Dijon, France), HCl was procured from Merck KGaA (Darmstadt, Germany), and other chemicals were sourced from Sigma-Aldrich Ltd. (Budapest, Hungary).

### 2.2. Radiochemistry, Partition Coefficient, and Metabolic Stability Measurements

5 mg (8.6 *μ*mol) c[KNGRE]-NH_2_ peptide was dissolved in 0.9 mL 0.1 mol/dm^3^ Na_2_CO_3_ buffer (pH = 8.42), then 7.5 mg (10.2 *μ*mol) NODAGA-NHS ester dissolved in 0.1 mL DMSO was added to the peptide. The reaction was mixed for 24 hours at room temperature. NODAGA-c(NGR) was purified with semipreparative HPLC (KNAUER) and freeze-dried. For the radiolabeling procedure, the *in vitro* serum stability, and the determination of log*p* value, the same protocol was used as it was described earlier by our research group [44].

Briefly, for the radiolabeling procedure, ^68^Ge/^68^Ga Obninsk-type generator (Cyclotron Co., Obninsk, Russia) was used. The peptide precursor was added to the buffered 68GaCl3 (in 0.1 M, 1 mL HCl, and 0.16 mL NaOAc), and it was incubated at 95°C for 5 min. After cooling down to room temperature, the mixture was pipetted on an OASIS® HLB 30 mg Cartridge (Waters, Milford, Massachusetts, USA), then it was resumed with physiological saline and filtrated to sterile form.

For the determination of the enzymatic stability, a stock solution was prepared from 100 *μ*L of ^68^Ga-NODAGA-c(NGR) solution and 1 mL PBS. 20 *μ*L from this solution was introduced to 480 *μ*L mouse serum (Sigma-Aldrich, St. Louis, Missouri, USA), and the mixture was tempered at 37°C. After 0, 30, 60, 90, and 120 min incubation time, 50 *μ*L sample was taken and 50 *μ*L abs. EtOH was added to the aliquots. Samples were centrifuged (20,000 rpm, 5 min), the supernatant was removed and diluted with the eluent of HPLC, then the analytical measurement was prepared [44].

The partition coefficient (log*p*) was determined using 1 : 1 mixture of octanol-PBS solution. As we described earlier, 10 *μ*L aqueous ^68^Ga-NODAGA-c(NGR) was added to the solution of 0.49 mL PBS and 0.5 mL 1-octanol. To reach the equilibrium state, the mixture was mixed firmly then was centrifuged (20,000 rpm, 5 min). 100 *μ*L samples were introduced into test tubes from each layer, and the radioactivity was determined with a calibrated gamma counter (Hewlett Packard Cobra II Gamma Counter) [44].

### 2.3. Cell Culturing

HT1080 (human fibrosarcoma) and B16-F10 cells (mouse melanoma) were purchased from ATCC (Virginia, USA) and cultured in GlutaMAX™ DMEM (Gibco™) supplemented with 1% (vol/vol) antimycotic and antibiotic solution (Gibco™) and 10% (vol/vol) heat-inactivated fetal bovine serum (Gibco™). The B16-F10 culture medium was further supplemented with 1% (vol/vol) MEM nonessential amino acid solution (Gibco™) and 1% (vol/vol) MEM vitamin solution (Gibco™). HT1080 and B16-F10 cells were cultured in 37°C, 5% CO_2_ atmosphere, and 95% humidity in a cell culture incubator (ESCO CCL-170B-8) using T75 flasks (Sarstedt Ltd., Hungary). For tumor induction, cells were used after five passages. The cell viability was verified with the trypan blue exclusion test.

### 2.4. Animal Housing

10-week-old male CB17 SCID (*n* = 30) and C57BL/6 (*n* = 30) mice were housed in a Sealsafe Blue line IVC system (Tecniplast, Akronom Ltd.) at a temperature of 26 ± 2°C with 55 ± 10% humidity and artificial lighting with a circadian cycle of 12 h. Sterile semisynthetic diet SDS VRF-1 (Animalab Ltd., Hungary) and sterile tap water were available ad libitum to all the animals. The animals received human care authorized by the Ethical Committee for Animal Research, University of Debrecen, Hungary. Experimental animals were kept and treated in compliance with all applicable sections of Hungarian Laws and directions and regulations of the European Union.

### 2.5. HT1080 and B16-F10 Tumor Induction

For experimental tumor induction, mice were anaesthetized with a dedicated small animal anaesthesia device (Tec3 Isoflurane Vaporizer, Eickemeyer Veterinary Equipment, UK) using 3% isoflurane (Forane, AbbVie), 0.4 L/min O_2_, and 1.4 L/min N_2_O. After depilation of the left shoulder area of the animals, CB17 SCID mice (*n* = 30) were injected subcutaneously with 1.5 × 10^6^ HT1080 (human fibrosarcoma) cells in 150 *μ*L (1/3 part of Matrigel and 2/3 part of saline), and C57BL/6 mice (*n* = 30) were injected subcutaneously with 3 × 10^6^ B16-F10 cells in 150 *μ*L of sterile saline. Tumor growth was assessed by caliper measurements by the same experienced researcher. The tumor size was calculated using the following formula: (largest diameter × smallest diameter^2^)/2.

### 2.6. Animal Treatment

Three days after tumor induction—at the tumor volume of approximately 52-55 mm^3^—HT1080 (*n* = 30) and B16-F10 (*n* = 30) tumor-bearing animals were randomly distributed into 3-3 groups as follows: control-untreated group (*n* = 10/tumor type), bestatin-treated group (*n* = 10/tumor type), and actinonin-treated group (*n* = 10/tumor type). For anticancer therapy, the mice of the bestatin-treated groups were injected intraperitoneally daily with 15 mg/kg bestatin dissolved in 150 *μ*L HumAqua for 7 days. For the actinonin-treated groups, 5 mg/kg actinonin (dissolved in 10 *μ*L abs. EtOH and diluted with 140 *μ*L HumAqua (aqua destillata, TEVA, Debrecen, Hungary)) was administrated daily by intraperitoneal injection for 7 days. The control-untreated group was injected intraperitoneally with 150 *μ*L saline for 7 days. The timescale of the experimental procedure is shown in Supplementary Material Fig. 1.

### 2.7. *In Vivo* PET Imaging and Image Analysis

Five and ten days after tumor cell implantation, control-untreated tumor-bearing and treated tumor-bearing animals were anaesthetized with 3% isoflurane and were injected with 5.5 ± 0.2 MBq APN/CD13-specific ^68^Ga-NODAGA-c(NGR) in 150 *μ*L saline via the lateral tail vein. After 90 min incubation time, 20 min static PET scans were performed from the tumorous area using the preclinical miniPET device (University of Debrecen, Faculty of Medicine, Department of Medical Imaging, Division of Nuclear Medicine and Translational Imaging). After 3D OSEM-LOR image reconstruction, volumes of interest (VOIs) were manually drawn around the examined regions using the BrainCAD image analysis software and quantitative standardized uptake values (SUVs) were calculated as follows: SUV = [VOI activity (Bq/mL)]/[injected activity (Bq)/animal weight (g)], assuming a density of 1 g/mL. Tumor-to-muscle (T/M) ratios were calculated from the activity of the tumor and background (muscle).

### 2.8. *Ex Vivo* Biodistribution Studies

After *in vivo* PET imaging, control-untreated tumor-bearing and treated tumor-bearing animals were euthanized with 5% Forane and dissected, and blood, urine, and samples were taken from the liver, spleen, kidney, small intestine, large intestine, stomach, heart, lungs, tumors, muscles, and fat. The weight and radioactivity of the selected organs and tissues were measured with a calibrated gamma counter (Hewlett Packard Cobra II Autogama Gamma Counter). The uptake of the APN/CD13-specific ^68^Ga-NODAGA-c(NGR) radiotracer was expressed as %ID/g tissue.

### 2.9. Western Blot Analysis

For western blot analysis, frozen tissue samples were pulverized under liquid nitrogen, and tissue homogenization was performed with TissueLyser II (QIAGEN). Tumors were lysed in a RIPA buffer. After protein isolation, protein samples (10-40 *μ*g) were separated on 10% SDS polyacrylamide gels and electrotransferred onto nitrocellulose membranes. After blocking, the membranes were incubated with primary anti-human and anti-mouse CD13 (from Santa-Cruz Biotechnology, Inc., USA) antibody at the dilution of 1 : 1000 overnight at 4°C. After washing, the membranes were probed with IgG HRP conjugated secondary antibody (Cell Signaling Technology, Inc., Beverly, MA, 1 : 2000). Beta-actin was used as a loading control. Bands were visualized by enhanced chemiluminescence reaction. Densitometry was performed using the ImageJ software. For the detailed protocol, please see Supplementary Material western blot analysis.

### 2.10. Statistical Analysis

Data were presented as mean ± SD of at least three independent experiments. The significance was calculated by Student's *t*-test (two-tailed), two-way ANOVA, and Mann-Whitney *U*-test. The significance level was set at *p* ≤ 0.05 unless otherwise indicated.

## 3. Results

### 3.1. Radiochemistry and Determination of the Partition Coefficient and Metabolic Stability

The conjugation of the APN/CD13-specific c(NGR) peptide with the bifunctional chelator NODAGA and the labeling procedures using ^68^Ga radionuclide were successfully completed. The radiochemical purity was always higher than 99% after the drug formulation process. The log*p* value of ^68^Ga-NODAGA-c(NGR) was −4.07 ± 0.1, which confirmed the highly hydrophilic property of the probe. The *in vitro* stability was determined in mouse serum at 37°C using the analytical radio-HPLC method. Samples were taken at different time points, and the ^68^Ga-NODAGA-c(NGR) remained stable after 120 min incubation time. The radiochemical purity of the tracer proved to be over 99%.

### 3.2. *In Vivo* PET Imaging of HT1080 Tumors

**Figure 1:**
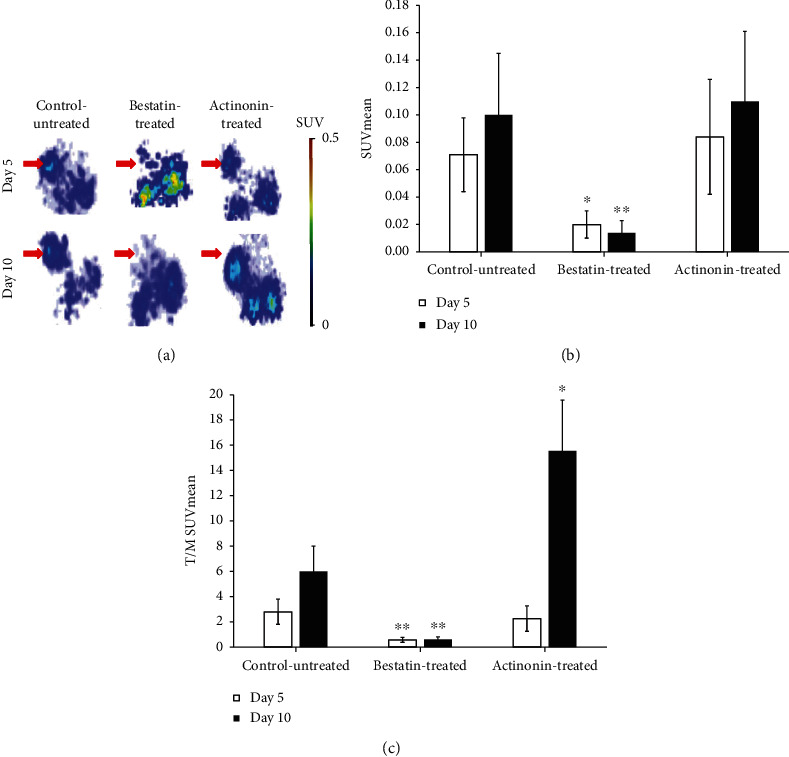
*In vivo* assessment of ^68^Ga-NODAGA-c(NGR) uptake of control-untreated and actinonin- and bestatin-treated HT1080 tumors. (a) Representative coronal decay-corrected PET images of control-untreated, bestatin-treated, and actinonin-treated tumor-bearing mice 5 (upper row) and 10 days (lower row) after tumor cell inoculation, and 90 min after intravenous injection of ^68^Ga-NODAGA-c(NGR). Red arrows: HT1080 tumor. (b) Quantitative decay-corrected SUV data analysis of ^68^Ga-NODAGA-c(NGR) uptake of treated and untreated HT1080 tumors 90 min after radiotracer injection. (c) Quantitative tumor-to-muscle ratio (T/M) analysis of treated and untreated HT1080 tumors 90 min after intravenous injection of ^68^Ga-NODAGA-c(NGR). SUVmean: standardized uptake value (mean). Significance levels: ^∗^*p* ≤ 0.05 and ^∗∗^*p* ≤ 0.01. Data is presented as mean ± SD.

The effect of actinonin and bestatin treatment on the APN/CD13 expression of HT1080 tumors was monitored by *in vivo* PET imaging studies. On days 3 (five days after tumor induction) and 7 (ten days after tumor induction) of the treatment, 20 min static PET scans were acquired from the tumorous area of control-untreated and treated HT1080 tumor-bearing mice 90 min after intravenous injection of ^68^Ga-NODAGA-c(NGR). After the qualitative PET image analysis, we found that the untreated-control tumors and actinonin-treated tumors were well identifiable at the investigated time points, indicating that actinonin treatment did not decrease the APN/CD13 expression in the tumors (Figure 1(a)). Interestingly, in the case of actinonin-treated tumors, ^68^Ga-NODAGA-c(NGR) accumulation was increased compared to the control-untreated tumors ten days after tumor cell inoculation. In contrast, in the bestatin-treated group, it was difficult to identify the HT1080 tumors by comparing to the untreated-control tumors due to the low ^68^Ga-NODAGA-c(NGR) accumulation, confirming the decreased expression of APN/CD13 (Figure 1(a), red arrows).

After the quantitative analysis of the reconstructed decay-corrected PET images, we found that the SUVmean values of the bestatin-treated tumors (0.02 ± 0.01) were significantly (*p* ≤ 0.05 and *p* ≤ 0.01) lower than that of the control-untreated tumors (0.07 ± 0.03) and the actinonin-treated tumors (0.08 ± 0.04) five days after HT1080 tumor cell implantation (Figure 1(b)). This significant difference persisted in the 10-day study, where the SUVmean values of the bestatin-treated, control-untreated, and actinonin-treated tumors were 0.01 ± 0.01, 0.10 ± 0.05, and 0.11 ± 0.05, respectively. By taking the SUVmean values, a moderate increase was observed in the untreated and actinonin-treated groups between 5 and 10 days. In contrast, there was a slight decrease in the SUVmean data in the bestatin-treated group, but these differences were not significant at *p* ≤ 0.05. Moreover, the accumulation of ^68^Ga-NODAGA-c(NGR) was higher (not significantly) in the actinonin-treated HT1080 tumors than in the untreated tumors (Figure 1(b)). This phenomenon was also recognizable in the PET images (Figure 1(a)). The contrast of the tumors (T/M SUVmean value) was also calculated in the control and treated groups, which determines the evaluability of the PET images and indicates the effectiveness of the treatment (Figure 1(c)). When the untreated and bestatin-treated HT1080 tumors were compared, significantly (*p* ≤ 0.01) lower T/M SUVmean values (0.57 ± 0.2 at day 5 and 0.61 ± 0.20 at day 10) were observed in the bestatin-treated group at each investigated time point. By analysing the actinonin-treated tumors, we found a lower T/M ratio (2.25 ± 1.0) five days after tumor cell inoculation, and a significantly (*p* ≤ 0.05) higher T/M ratio was observed (15.57 ± 4.0) at day 10, than that of the untreated tumors, where the T/M ratios were 2.8 ± 1.0 and 6.0 ± 2.0 at days 5 and 10, respectively (Figure 1(c)). These results suggest that bestatin is a suitable and effective APN/CD13 inhibitor in HT1080 experimental tumors.

### 3.3. *In Vivo* PET Imaging of B16-F10 Melanoma Tumors

**Figure 2:**
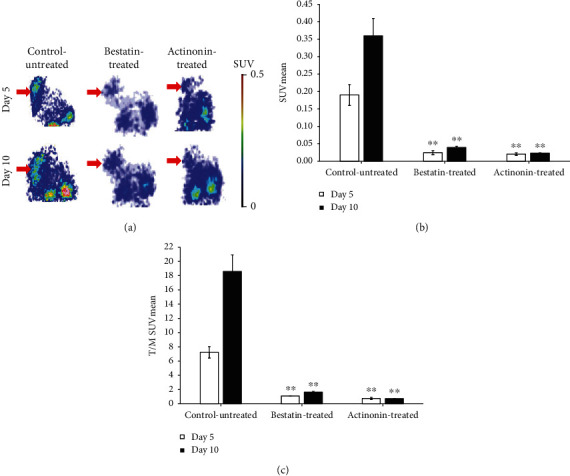
*In vivo* assessment of ^68^Ga-NODAGA-c(NGR) uptake of control-untreated and actinonin- and bestatin-treated B16-F10 tumors. (a) Representative coronal decay-corrected PET images of control-untreated, bestatin-treated, and actinonin-treated B16-F10 tumor-bearing C57BL/6J mice 5 (upper row) and 10 days (lower row) after tumor cell inoculation and 90 min after intravenous injection of ^68^Ga-NODAGA-c(NGR). Red arrows: B16-F10 tumor. (b) Quantitative decay-corrected SUV data analysis of ^68^Ga-NODAGA-c(NGR) uptake of treated and untreated B16-F10 tumors 90 min after radiotracer injection. (c) Quantitative tumor-to-muscle ratio (T/M) analysis of treated and untreated B16-F10 tumors 90 min after intravenous injection of ^68^Ga-NODAGA-c(NGR). SUVmean: standardized uptake value (mean). Significance level: ^∗∗^*p* ≤ 0.01. Data is presented as mean ± SD.

For the assessment of the effect of actinonin and bestatin treatment on the APN/CD13 expression of B16-F10 tumors, PET scans were performed 90 min after the intravenous injection of ^68^Ga-NODAGA-c(NGR) five and ten days after tumor induction (Figure 2). High ^68^Ga-NODAGA-c(NGR) accumulation was observed in the subcutaneously growing B16-F10 melanoma tumors by qualitative PET image analysis. The APN/CD13 expression was clearly visualized in the untreated group due to the high specificity of the radiotracer; furthermore, moderate radiotracer uptake was observed in the bestatin- and actinonin-treated groups (Figure 2(a), red arrows).

By the quantitative image analysis of the decay-corrected PET images at day 5, significantly (*p* ≤ 0.01) lower SUVmean values were observed in the actinonin-treated (0.02 ± 0.001) and bestatin-treated groups (0.03 ± 0.01) in comparison with the control-untreated group, where elevated SUVmean (0.19 ± 0.03) values were measured (Figure 2(b)). This significant difference was also observed on the 10^th^ day, when the SUVmean values of actinonin-treated, bestatin-treated, and control-untreated groups were 0.02 ± 0.001, 0.04 ± 0.001, and 0.36 ± 0.05, respectively. In the control-untreated group, a remarkable increasing of SUVmean values was seen between the 5^th^ and 10^th^ days; furthermore, negligible elevation was observed in the bestatin- and actinonin-treated groups at the same time points, but these differences were not significant at *p* ≤ 0.05. In the control-untreated group, considerably increased T/M SUVmean values were seen between days 5 (7.24 ± 0.80) and 10 (18.60 ± 2.32). In addition, significantly (*p* ≤ 0.01) lower T/M ratios were observed on days 5 and 10 in the actinonin-treated group (0.75 ± 0.15 at day 5 and 0.74 ± 0.05 at day 10) and in the bestatin-treated group (1.11 ± 0.01 and 1.63 ± 0.09 at days 5 and 10, respectively) (Figure 2(c)).

### 3.4. *Ex Vivo* Biodistribution Studies

**Figure 3:**
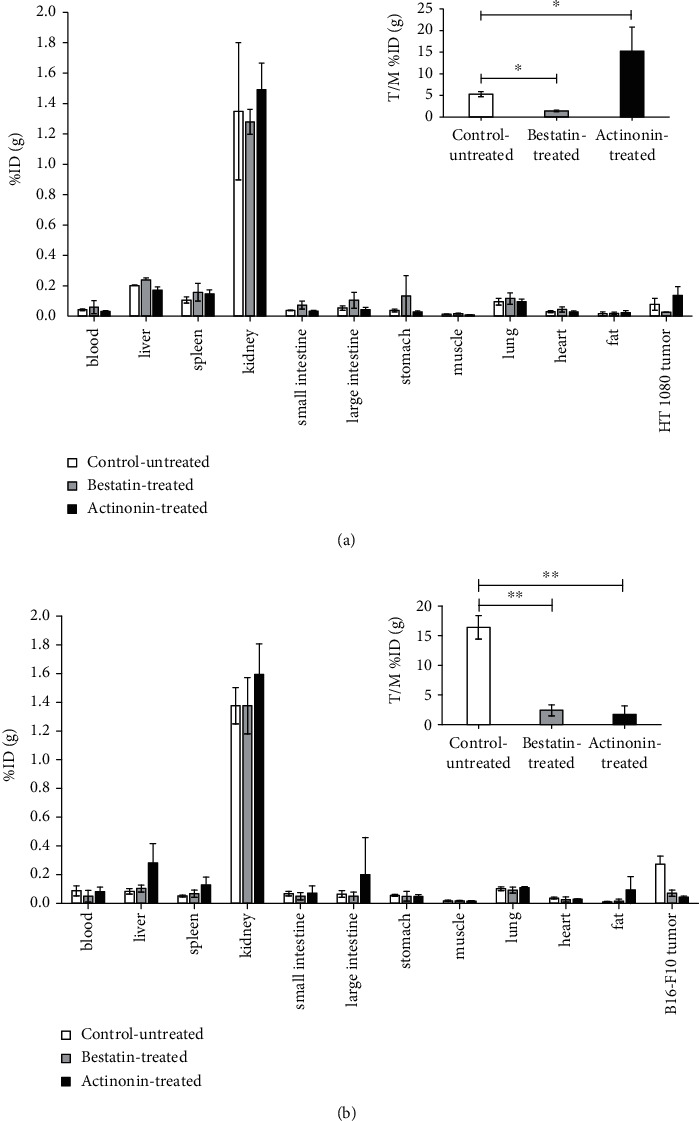
*Ex vivo* biodistribution studies of ^68^Ga-NODAGA-c(NGR) in control-untreated, bestatin-treated, and actinonin-treated HT1080 (a) and B16-F10 (b) tumor-bearing mice. Inserts: T/M (%ID/g tumor/%ID/g muscle) ratios. Significance levels: ^∗^*p* ≤ 0.05 and ^∗∗^*p* ≤ 0.01.

For evaluating the effect of bestatin and actinonin treatment on the expression of APN/CD13 of HT1080 and B16-F10 tumors, *ex vivo* biodistribution studies were performed after *in vivo* PET imaging. In treated and untreated HT1080 and B16-F10 tumor-bearing animals, high ^68^Ga-NODAGA-c(NGR) accumulation was observed in the kidneys (ID%/g approx. 1.3-1.6), and moderate uptake was observed in the liver (ID%/g approx. 0.08-0.28). Relatively low radiotracer accumulation was noticed in the investigated thoracic and abdominal organs and tissues (Figures 3(a) and 3(b)). No significant differences (at *p* ≤ 0.05) were found in the ^68^Ga-NODAGA-c(NGR) uptake between healthy organs and tissues when the untreated and treated animals were compared. By analysing the subcutaneously growing tumors, we found that the *ex vivo* %ID/g and T/M %ID/g values correlated well with *in vivo* SUVmean values in both tumor types. Similarly, to the *in vivo* PET imaging results, we found significantly (*p* ≤ 0.05) lower radiopharmaceutical uptake after bestatin treatment and higher radiotracer accumulation in the actinonin-treated HT1080 tumors (Figure 3(a)). In contrast, significantly lower (*p* ≤ 0.01) ^68^Ga-NODAGA-c(NGR) accumulation was observed in both bestatin and actinonin-treated B16-F10 melanoma tumors compared to the untreated-control tumors (Figure 3(b)). The detailed *ex vivo* data are shown in Supplementary Material Table 1.

### 3.5. Impact of Bestatin and Actinonin Treatment on Tumor Volume

**Figure 4:**
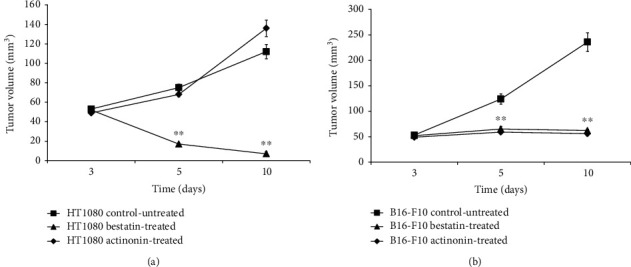
Impact of bestatin and actinonin treatment on tumor growth. Treatment began 3 days after HT1080 and B16-F10 tumor cell inoculation at the tumor volume of approx. 52-55 mm^3^. (a) Impact of treatment on HT1080 tumors (*n* = 10/group). (b) Impact of treatment on B16-F10 melanoma tumors (*n* = 10/group). Significance level: ^∗∗^*p* ≤ 0.01.

Three days after the subcutaneous injection of HT1080 and B16-F10 tumor cells, mice were treated with intraperitoneal injection of bestatin or actinonin for seven days. Tumor-bearing control-untreated mice were injected with saline. In the HT1080 tumor-bearing groups, the volumes of the control-untreated tumors were 53.2 ± 2.3 at day 3, 75.2 ± 3.5 at day 5, and 112 ± 7.36 at day 10; the actinonin-treated tumors also showed an increased tumor size with the volumes of 49.1 ± 2.0, 68.08 ± 2.5, and 136.0 ± 8.5 at days 3, 5, and 10, respectively. In contrast, there was a significant (*p* ≤ 0.01) reduction in the size of tumors after bestatin treatment (52.3 ± 2.8 at day 3, 17 ± 1.2 at day 5, and 7.0 ± 2.0 at day 10) (Figure 4(a)). In the B16-F10 tumor-bearing groups, the size of tumors in the treated groups showed a negligible increase after the treatment. The tumor volumes of the bestatin-treated group were 52.1 ± 2.6 at day 3, 65.0 ± 5.2 at day 5, and 62.0 ± 2.8 at day 10, and in the case of the actinonin-treated group, the tumor volumes were 49.0 ± 2.7, 59 ± 4.9, and 56.1 ± 2.1 at days 3, 5, and 10, respectively. In contrast, the size of control tumors showed a significant increase (Figure 4(b)).

### 3.6. Western Blot Analysis

**Figure 5:**
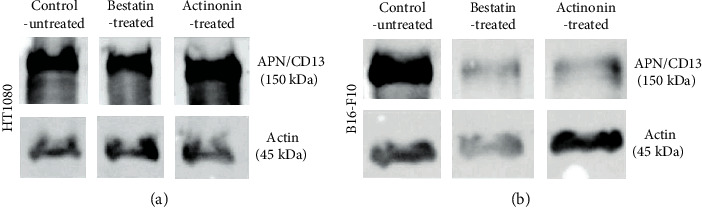
Qualitative western blot analysis of APN/CD13 expression in subcutaneously transplanted untreated and treated HT1080 (a) and B16-F10 (b) tumors.

The expression of APN/CD13 was verified by western blot analysis in subcutaneous transplanted HT1080 and B16-F10 tumors (Figure 5). We found no significant differences between the amounts of APN/CD13 protein in the control-untreated tumors and in the treated HT1080 tumors. In contrast, the expression of the investigated neoangiogenic marker in B16-F10 melanoma after bestatin or actinonin treatment was significantly lower compared to the untreated melanoma tumor, which showed strong positivity (Figure 5(b) and Supplementary Material Fig. 2).

## 4. Discussion

It has been reported that APN/CD13 is overexpressed in several solid tumors (e.g., malignant melanoma, mesenchymal tumors, gynecological cancers, colorectal cancers, renal cancers, non-small cell lung cancer, and gastric carcinoma) and plays an important role in tumor-associated neoangiogenesis, tumorigenesis, and development of metastasis [29, 46]. As a result, it is an important diagnostic and prognostic marker and a promising target for antitumor therapy in both clinical and preclinical research; moreover, this ectopeptidase is also a potential target in the field of positron emission tomography (PET) due to the fact that radiolabeled NGR peptide sequences specifically bind to APN/CD13 [44, 45]. Beyond diagnostic applications, several antitumor therapies are known from the literature that act through the direct inhibition of the APN/CD13 molecule, and also various NGR-conjugated therapeutic agents have been developed that deliver different cytotoxins (e.g., NGR-coated liposomes, doxorubicin-NGR conjugates, and NGR-TNF alpha conjugates) to APN/CD13-positive solid tumors [29].

Two main types of APN/CD13 inhibitors are known, the synthetic and the naturally occurring form. Molecules of both types generally have a zinc-binding group and are effective in the inhibition of tumor angiogenesis and cell migration. The naturally occurring APN/CD13 inhibitors are originating from bacterial cultures; accordingly, bestatin originates from *Streptomyces olivoreticuli* and actinonin—as an antibiotic derivative of L-prolinol—was isolated from *Streptomyces cutter C*/2 [30, 43]. The antitumor activity of these naturally occurring APN/CD13 inhibitors is mediated by several, often independent mechanisms. Actinonin and bestatin have immunomodulatory and host-mediated antitumor activities, and by binding to the zinc domains of APN/CD13 in cancer cells, they inhibit the function of this exopeptidase. In contrast, it was found that, e.g., actinonin also inhibited the growth of CD13-negative tumor cells, suggesting that the antitumor effect is not only mediated by cell surface APN/CD13. It was observed that the effect of these inhibitors was mediated partly through the inhibition of other zinc-dependent intracellular enzymes resulting in cell cycle G1 arrest and apoptosis. Among the direct inhibitors, bestatin inhibits at least 12 different aminopeptidases, as these exopeptidases—including APN/CD13—generally have a diverse and overlapping substrate specificity. Despite the fact that these inhibitors are not specific for only one type of APN, because of their strong antitumor activity, several APN inhibitors have entered clinical trials, e.g., bestatin (ubenimex) [29, 34, 35, 43].

In this present study, the effect of bestatin and actinonin treatment on APN/CD13 expression of HT1080 and B16-F10 tumors was investigated and followed by using APN/CD13-specific ^68^Ga-NODAGA-c(NGR) radiopharmaceutical and *in vivo* PET imaging.

For the *in vivo* assessment of the antitumor effect of bestatin and actinonin treatment, HT1080 and B16-F10 cell lines were used to establish tumor-bearing animals. Previous studies have shown that these cell lines showed APN/CD13 positivity by western blot analysis, flow cytometry, immunofluorescence analysis, immunohistochemistry, RT-PCR, and optical imaging [19, 26, 39, 47]. Our *in vivo* PET imaging results (Figures 1(a) and 2(a)) correlated well with these *in vitro* findings where the control-untreated HT1080 and B16-F10 tumors were clearly visualized by the APN/CD13-specific ^68^Ga-NODAGA-c(NGR) radiopharmaceutical. The western blot analysis also confirmed the strong APN/CD13 positivity in the investigated tumors (Figure 5).

In our further *in vivo* experiments, the efficacy of the actinonin and bestatin treatments was investigated on experimental tumors. The antitumor effect of actinonin was investigated earlier by Xu and coworkers [43] on human (HL60, NB4) and murine (AKR) leukemia and lymphoma (RAJI, DAUDI) cell lines. In these *in vitro* studies, actinonin blocked the tumor growth of APN/CD13-positive leukemia and arrested the growth of APN/CD13-negative lymphomas interestingly. The authors supposed that the antitumor effect of actinonin is not derived only by the inhibition of APN/CD13. In *in vivo* studies, syngeneic AKR tumor-bearing mice were treated with actinonin at the dose of 5 mg/kg, which generated a full antitumor effect; however, this action was not seen in subcutaneous human lymphoma-bearing nude mice. In our *in vivo* study, no decrease of APN/CD13 expression in HT1080 tumors was observed by PET imaging after actinonin treatment. Furthermore, actinonin did not block the subcutaneously transplanted HT1080 tumor growing in CB17 SCID mice. In contrast, actinonin successfully exerted its antitumor effect in B16-F10 melanoma tumors growing in C57BL/6 mice (Figures 1 and 4). As Xu and coworkers [43] mentioned, one possible explanation for this phenomenon is that these inhibitors (such as bestatin or actinonin) may act as an immunomodulator; hence, they do not have significant growth-inhibiting properties for tumors in an immunosufficient mouse (CB17 SCID). Another possible explanation is the dosage of actinonin: 5 mg/kg was enough to block the growth of syngeneic mouse leukemia, but in human fibrosarcoma, the dose used was not sufficient to produce the antitumor effect. Presumably, the dose sensitivity of various types of tumors is different; dose-dependent studies are required [43].

In our studies, when the efficacy of bestatin was investigated, we found that this competitive inhibitor of APN/CD13 exerted its antitumor effect in both investigated tumors, where the tumor size and the APN/CD13 receptor expression decreased compared to control-untreated tumors (Figures 1 and 4). Due to the fact that APN/CD13 can be found on several tumors and immune cells (e.g., T and B cell precursors, monocytes, and dendritic cells) [16, 48], bestatin can directly inhibit tumor growth and angiogenesis by blocking APN/CD13 on the tumorous cell surface; furthermore, bestatin can modulate the antitumoral immune response. Bestatin has numerous effects on the immune system, either *in vitro* or *in vivo*. Bestatin can enhance cell-mediated immunity through inducing a blastogenic effect on T cells [49–51] and can activate macrophages [49, 51]. Ishizuka and coworkers [51] reported that a high dose of bestatin has a mitogenic effect on B lymphocytes as well. Bestatin can facilitate the differentiation of T cell precursors into CD4+ T helper cells [52]. Abe and coworkers [53] reported that bestatin enhanced bone marrow stem cells; thus, it can be effective with other cytotoxic agents in the inhibition of tumor growth. Amoscato and coworkers [54] described that bestatin may increase or reduce the NK cell activity in CB17 SCID and C57BL/6 mice, which was influenced by the dose and the administration route. This can be an explanation for the effect of bestatin on both HT1080 and B16-F10 cell lines. Moreover, it was an interesting observation by western blot analysis that although bestatin inhibited the tumor growth and ^68^Ga-NODAGA-c(NGR) uptake in the HT1080 tumor model, this APN/CD13 competitive inhibitor did not significantly reduce the amount of APN/CD13 protein in HT1080 tumors (Figure 5(a)). This may be explained by the dose used and the period of treatment in this study, which reduced the functionality and enzymatic activity of APN/CD13, but not the protein expression. In addition, naturally occurring APN/CD13 inhibitors are potential candidates for antimetastasis and antiangiogenesis therapy; however, more and more pathways are being discovered in which APN/CD13 plays a significant role that may complicate the development of anticancer drugs for APN/CD13.

## 5. Conclusion

Based on our *in vivo* results, we concluded that due to the significant reduction of the ^68^Ga-NODAGA-c(NGR) uptake in the investigated tumors after bestatin treatment, this APN/CD13 inhibitor might be suitable for suppressing the neoangiogenic process and APN/CD13 activity of experimental HT1080 and B16-F10 tumors. We also confirmed that ^68^Ga-NODAGA-c(NGR) is an applicable radiotracer for the *in vivo* monitoring of the efficacy of the APN/CD13 inhibition-based anticancer therapies.

## Data Availability

The data used to support the findings of this study are available from the corresponding author upon request.

## Abbreviations

APN/CD13: Aminopeptidase N (CD13)
c(KNGRE)-NH_2_: Cyclic(lysyl-asparaginyl-glycyl-argininyl-glutamic acid amide)
NGR: Asparaginyl-glycyl-arginine peptide sequence
NODAGA: 1,4,7-Triazacyclononane,1-glutaric acid-4,7-acetic acid
NODAGA-c(NGR): c[Lys(NODAGA)-Asn-Gly-Arg-Glu]-NH_2_
PET: Positron emission tomography
sc: Subcutaneous
SUV: Standardized uptake value
SUVmean: Standardized uptake value (mean)
T/M: Tumor-to-muscle ratio.

## Supplementary Materials

Supplementary Materials Supplementary data 1: timescale of the experimental procedure (Figure 1). Supplementary data 2: *ex vivo* biodistribution data (%ID/g values) of control and treated subcutaneous HT1080 and B16-F10 tumor-bearing animals (Table 1). Supplementary data 3: detailed methods and results of the western blot analysis (Figure 2).
