# Supplementary material for: In Vivo Molecular Imaging of the Efficacy of Aminopeptidase N (APN/CD13) Receptor Inhibitor Treatment on Experimental Tumors Using 68Ga-NODAGA-c(NGR) Peptide
Source: Biomed Res Int. 2021 Mar 10;2021:6642973. doi: 10.1155/2021/6642973 (PMC7972841; doi:10.1155/2021/6642973)
Supplement: Supplementary Materials — Supplementary data 1: timescale of the experimental procedure (Figure 1). Supplementary data 2: ex vivo biodistribution data (%ID/g values) of control and treated subcutaneous HT1080 and B16-F10 tumor-bearing animals (Table 1). Supplementary data 3: detailed methods and results of the western blot analysis (Figure 2). [file 6642973.f1.docx]

**Supplementary material**

***In vivo* molecular imaging of the efficacy of aminopeptidase N (APN/CD13) receptor inhibitor treatment on experimental tumors using ^68^Ga-NODAGA-c(NGR) peptide**

Adrienn Kis^1,2^, Noémi Dénes^1,3^, Judit P. Szabó^1,2^, Viktória Arató^1^, Lívia Beke^4^, Orsolya Matolay^4^, Kata Nóra Enyedi^5^, Gábor Méhes^4^, Gábor Mező^5,6^, Péter Bai^7,8,9^, István Kertész^1^, György Trencsényi^1,2,3,*^

^1^Division of Nuclear Medicine and Translational Imaging, Department of Medical Imaging, Faculty of Medicine, University of Debrecen, Nagyerdei St. 98, H-4032 Debrecen, Hungary;

^2^Doctoral School of Clinical Medicine, Faculty of Medicine, University of Debrecen, Nagyerdei St. 98, H-4032 Debrecen, Hungary

^3^Gyula Petrányi Doctoral School of Allergy and Clinical Immunology, Faculty of Medicine, University of Debrecen, Nagyerdei St. 98, H-4032 Debrecen, Hungary

^4^Department of Pathology, Faculty of Medicine, University of Debrecen, Nagyerdei St. 98, H-4032 Debrecen, Hungary

^5^Eötvös Loránd University, Faculty of Science, Institute of Chemistry, Budapest, Hungary

^6^MTA-ELTE, Research Group of Peptide Chemistry, Hungarian Academy of Sciences, Eötvös L. University, Budapest, Hungary

^7^Department of Medical Chemisty, University of Debrecen, Nagyerdei St. 98, H-4032 Debrecen, Hungary;

^8^MTA-DE Lendület Laboratory of Cellular Metabolism, Debrecen, Hungary;

^9^Research Center for Molecular Medicine, University of Debrecen, Nagyerdei St. 98, H-4032 Debrecen, Hungary.

* corresponding author

Corresponding author:

György Trencsényi, PhD; Division of Nuclear Medicine and Translational Imaging, Department of Medical Imaging, Faculty of Medicine, University of Debrecen, Nagyerdei St. 98, H-4032 Debrecen, Hungary

E-mail: trencsenyi.gyorgy@med.unideb.hu

**Supplementary material**

**Supplementary data 1**

**Fig. 1** Timescale of the experimental procedure.


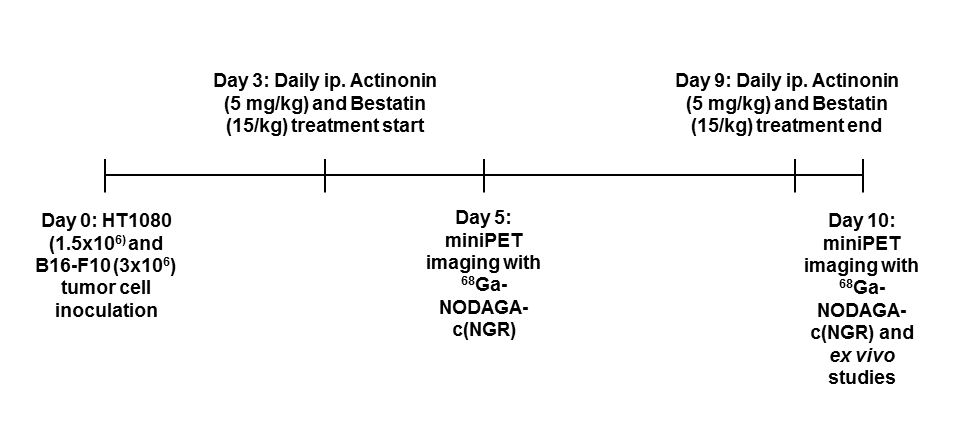


**Supplementary data 2**

**Table 1.** *Ex vivo* biodistribution data (%ID/g values) of control and treated subcutan HT1080 and B16-F10 tumor-bearing animals. %ID/g values are presented as mean±SD.

| **Organ or tissue name** | **HT1080 tumor** | | | **B16-F10 tumor** | | |
| --- | --- | --- | --- | --- | --- | --- |
|  | **Control-untreated (n=10)** | **Bestatin-treated (n=10)** | **Actinonin-treated (n=10)** | **Control-untreated (n=10)** | **Bestatin-treated (n=10)** | **Actinonin-treated (n=10)** |
| **blood** | 0.04±0.01 | 0.06±0.04 | 0.03±0.00 | 0.09±0.03 | 0.05±0.04 | 0.08±0.03 |
| **liver** | 0.20±0.01 | 0.24±0.01 | 0.17±0.02 | 0.08±0.02 | 0.10±0.02 | 0.28±0.13 |
| **spleen** | 0.11±0.02 | 0.16±0.06 | 0.15±0.03 | 0.05±0.01 | 0.07±0.03 | 0.13±0.05 |
| **kidney** | 1.35±0.45 | 1.28±0.08 | 1.49±0.18 | 1.38±0.13 | 1.38±0.20 | 1.59±0.21 |
| **small intestine** | 0.04±0.00 | 0.07±0.03 | 0.03±0.00 | 0.07±0.01 | 0.05±0.02 | 0.07±0.05 |
| **large intestine** | 0.06±0.01 | 0.11±0.05 | 0.04±0.01 | 0.06±0.02 | 0.05±0.03 | 0.20±0.26 |
| **stomach** | 0.04±0.01 | 0.14±0.13 | 0.03±0.00 | 0.06±0.01 | 0.05±0.03 | 0.05±0.01 |
| **muscle** | 0.01±0.00 | 0.02±0.00 | 0.01±0.00 | 0.02±0.00 | 0.02±0.00 | 0.02±0.00 |
| **lung** | 0.10±0.02 | 0.12±0.04 | 0.10±0.02 | 0.10±0.01 | 0.09±0.02 | 0.11±0.01 |
| **heart** | 0.03±0.01 | 0.05±0.02 | 0.03±0.01 | 0.04±0.01 | 0.03±0.02 | 0.03±0.00 |
| **fat** | 0.02±0.01 | 0.02±0.01 | 0.03±0.01 | 0.01±0.00 | 0.02±0.01 | 0.09±0.09 |
| **tumor** | 0.08±0.04 | 0.03±0.00 | 0.14±0.06 | 0.27±0.05 | 0.07±0.02 | 0.04±0.01 |
| **T/M ID%/g** | 5.30±0.56 | 1.45±0.15 | 15.27±5.52 | 16.38±1.97 | 2.40±0.92 | 1.71±1.45 |

**Supplementary data 3**

**Western blot analysis**

For western blot analysis frozen tissue samples were pulverized under liquid nitrogen and tissue homogenization was performed with TissueLyser II (QIAGEN). Cells were lysed in RIPA buffer (50 mM Tris, 150 mM NaCl, 0.1 % SDS, 1 % TritonX 100, 0.5 % sodium deoxycolate, 1 mM EDTA, 1 mM Na3VO4, 1 mM NaF, 1 mM PMSF, protease inhibitor cocktail). After tissue homogenization, the samples were subjected to protein isolation. Protein samples (10-40 µg) were separated on 10% SDS polyacrylamide gels and electrotransferred onto nitrocellulose membranes. After blocking for 1 h with TBST containing 5 % BSA, the membranes were incubated with primary anti-human and anti-mouse CD13 (from Santa-Cruz Biotechnology Inc. USA) antibody at the dilution of 1:1000 overnight at 4 °C. After washing with 1 x TBST solution, the membranes were probed with IgG HRP conjugated secondary antibody (Cell Signaling Technology, Inc. Beverly, MA, 1:2000). Bands were visualized by enhanced chemiluminescence reaction (SuperSignal West Pico Solutions, Thermo Fisher Scientific Inc., Rockford, USA). Densitometry was performed using the Image J software. Upon densitometry, negative control samples (mouse large intestine) were considered to be 1 and values are expressed as fold change relative to controls to reduce unwanted variations. Beta-actin was used as a loading control, and mouse kidney was used as positive control.


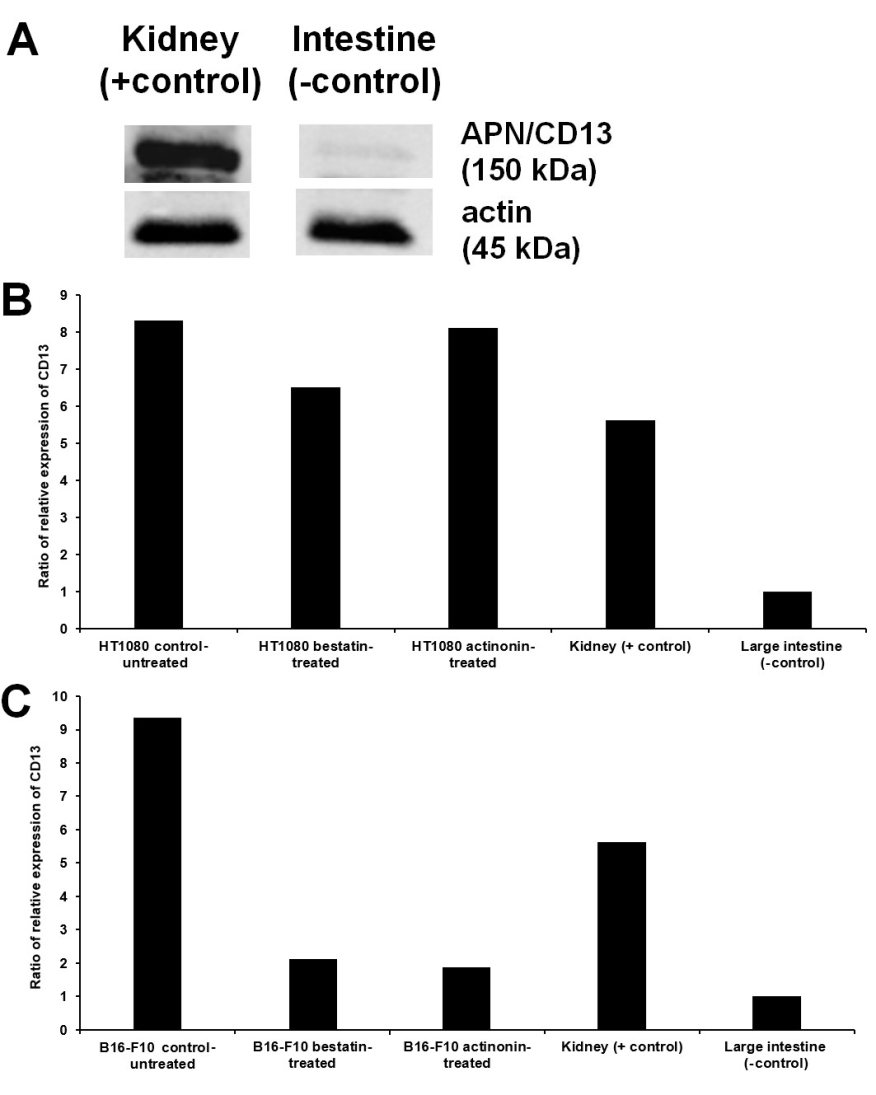


**Fig. 2** Qualitative analysis of the APN/CD13 expression of the positive and negative controls (A) and the qualitative western blot analysis of APN/CD13 expression in subcutaneously transplanted untreated and treated HT1080 (B) and B16-F10 (C) tumors. In densitometry, negative control samples (mouse large intestine) were considered to be 1 and values are expressed as fold change relative to controls.
